# Elucidating the role of circNFIB in myocardial fibrosis alleviation by endogenous sulfur dioxide

**DOI:** 10.1186/s12872-022-02909-x

**Published:** 2022-11-20

**Authors:** Jia Liu, Ranran Zhang, Dahai Wang, Yi Lin, Cui Bai, Nana Nie, Shan Gao, Qiuye Zhang, Hong Chang, Chongmin Ren

**Affiliations:** 1 Department of pediatric nephrology and rheumotology, The Affiliated Hospital of Qingdao University, Qingdao, China; 2 Department of orthopedic oncology, The Affiliated Hospital of Qingdao University, Qingdao, China

**Keywords:** Myocardial fibrosis, Sulfur dioxide, Aspartate aminotransferase, circNFIB, Wnt, β-catenin, p38 MAPK

## Abstract

**Background:**

To investigate the role of circNFIB in the alleviation of myocardial fibrosis by endogenous sulfur dioxide (SO_2_).

**Methods:**

We stimulated cultured neonatal rat cardiac fibroblasts with transforming growth factor-β1 (TGF-β1) and developed an in vitro myocardial fibrosis model. Lentivirus vectors containing aspartate aminotransferase 1 (AAT1) cDNA were used to overexpress AAT1, and siRNA was used to silence circNFIB. The SO_2_, collagen, circNFIB, Wnt/β-catenin, and p38 MAPK pathways were examined in each group.

**Results:**

In the in vitro TGF-β1-induced myocardial fibrosis model, endogenous SO_2_/AAT1 expression was significantly decreased, and collagen levels in the cell supernatant and type I and III collagen expression, as well as α-SMA expression, were all significantly increased. TGF-β1 also significantly reduced circNFIB expression. AAT1 overexpression significantly reduced myocardial fibrosis while significantly increasing circNFIB expression. Endogenous SO_2_ alleviated myocardial fibrosis after circNFIB expression was blocked. We discovered that circNFIB plays an important role in the alleviation of myocardial fibrosis by endogenous SO_2_ by inhibiting the Wnt/β-catenin and p38 MAPK pathways.

**Conclusion:**

Endogenous SO_2_ promotes circNFIB expression, which inhibits the Wnt/β-catenin and p38 MAPK signaling pathways, consequently alleviating myocardial fibrosis.

**Supplementary Information:**

The online version contains supplementary material available at 10.1186/s12872-022-02909-x.

## Background

Myocardial fibrosis refers to quantitative and qualitative changes in the collagen network in the interstitial myocardium; these changes are caused by myocardial ischemic injury, systemic disease, drugs, or any other harmful stimulation that affects the circulatory system or heart itself [1]. Myocardial fibrosis can alter the structure of the heart, promote the occurrence of cardiac dysfunction, induce arrhythmia, and eventually lead to refractory heart failure, which is the main cause of death in patients with cardiac diseases. Considering that myocardial fibrosis seriously affects the clinical treatment and prognosis of patients [2, 3], its prevention, delay, and/or reversal are pivotal for treating heart diseases [4].

Endogenous sulfur dioxide (SO_2_), a newly discovered gaseous signaling molecule, is involved in cardiovascular function regulation and is associated with dilating blood vessels, inhibiting inflammation, and improving vascular collagen remodeling [5–7]. Furthermore, SO_2_ has been reported to promote collagen degradation and reduce collagen deposition in rat pulmonary artery fibroblasts by inhibiting the transforming growth factor-β1 (TGF-β1)/Smad signaling pathway [8]. Some studies have reported that SO_2_ inhibits cardiac fibroblast (CF) proliferation and migration to inhibit myocardial fibrosis. However, the precise mechanism and specific target of SO_2_/aspartate aminotransferase 1 (AAT1, the key enzyme for endogenous SO_2_ production) in alleviating myocardial fibrosis need to be further explored; doing so should provide strong theoretical support for the clinical exploration of effective therapeutic targets.

Circular RNAs (circRNAs) are noncoding RNAs that are present in all organisms and participate in various physiological and pathological biological processes in humans [9]. CircRNAs have been shown to regulate cardiomyocyte proliferation, apoptosis, and collagen production via different mechanisms, thereby affecting myocardial fibrosis and participating in the occurrence and development of diverse cardiovascular diseases [10]. A study recently reported that circNFIB expression was significantly decreased in mouse postmyocardial infarction heart samples and in TGF-β-treated primary adult CFs [11]. However, the relationship between endogenous SO_2_ and noncoding RNAs has not been studied.

In this study, we aimed to examine whether SO_2_/AAT1 can alleviate myocardial fibrosis by regulating circNFIB expression. Our goal was to identify a possible diagnostic and therapeutic target for the treatment of myocardial fibrosis with SO_2_/AAT1.

## Methods

### Isolation and culture of neonatal rat CFs

Neonatal rats were soaked in 75% alcohol for 5 min and transferred to an ultraclean table. After being thoroughly disinfected, the heart tissue was collected and washed with sterile PBS in a Petri dish. Subsequently, the tissue was transferred into a culture dish containing 75% alcohol, soaked for 15 s to kill most heart capsule cells and then immediately transferred into a culture dish containing PBS for concussion cleaning. The aortic arch and atrial tissue were removed using scissors and tweezers; the ventricular part was reserved as much as possible, cut into pieces after being cleaned with PBS and predigested for 10 min at 37 °C in 0.25% trypsin. Subsequently, fetal bovine serum was added to terminate the digestion. After being cleaned with PBS several times, the pieces were evenly inoculated on the surface of the culture bottle, and Dulbecco’s Modified Eagle Medium (DMEM, Gibco, 11,965,092) containing 10% fetal bovine serum was added. The bottle was then placed in a 37 °C carbon dioxide incubator for 2–4 h. Adherent cells (mainly CFs) were further cultured in culture medium. Unattached cells in suspension were cultured for 1-1.5 h under the same conditions, and then adherent cells were collected. According to the difference in adherence time between myocardial cells and CFs, the adhesion speed of fibroblasts is faster. Therefore, CFs were obtained by the differential adhesion method. After 2–4 days, fibroblasts climbed out around the tissue blocks. The cells were fed every 2–3 days and passaged once they reached approximately 70-80% confluence. Cells at passage 3 were collected and used for further experiments.

### TGF-β1 stimulation

CFs in the logarithmic phase were collected, and recombinant TGF-β1 (HZbscience, ZY124Bo011, 5 ng/mL) was added, followed by incubation for 24 h. This in vitro myocardial fibrosis model was established after long-term research and with reference to published studies [11, 12].

### AAT1 overexpression and circNFIB silencing

The AAT1 overexpression vector and circNFIB silencing sequence were purchased from Shanghai GenePharma Co., Ltd. AAT1 was overexpressed using a lentivirus vector containing AAT1 cDNA, and circNFIB was silenced with siRNA. The target sequence of si-circNFIB was 5’-GAGATCAAGCACCCATAAC-3’, and a scrambled siRNA sequence was used as the control (5’-TTCTCCGAACGGTCACGT-3’). Lentiviruses or liposomes were used to transfer the corresponding vectors into CFs according to the manufacturer’s instructions.

### High-performance liquid chromatography measurement of SO_2_ production in CFs

High-performance liquid chromatography (Agilent Technologies, Agilent 1100,) was performed to determine the SO_2_ concentration in the culture supernatant. The culture supernatant (100 µL) was aspirated into a clean tube; 70 µL of 0.212 M sodium borohydride in 0.05 M Tris-HCl (pH 8.5) was added to the supernatant, followed by incubation for 30 min at room temperature. Subsequently, 5 µL of 70 mM monobromobimane in acetonitrile was added to the solution and incubated for 10 min at 42 °C. After 40 µL of 1.5 M perchloric acid was added and thorough mixed by vortexing, the solution was centrifuged at 12,400 × g for 10 min to remove protein precipitates. The supernatant was gently mixed with 10 µL of 2.0 M Tris-HCl for neutralization and then centrifuged again at 12,400 × g for 10 min. All centrifugation steps were performed at room temperature. The supernatant (100 µL) that was finally obtained was transferred into foil-wrapped tubes, and 10 µL of this supernatant was injected into a high-performance liquid chromatography column. The column was equilibrated with a buffer composed of methanol, acetic acid, and water (5.00:0.25:94.75 *v/v*, pH 3.4). The sulfite-bimane adduct was detected with excitation at 392 nm and emission at 479 nm.

### Collagen determination

Total soluble collagen was measured in the culture supernatant by the Sircol Soluble Collagen Assay (Biocolor, S1000). Briefly, 50 µL of cell culture supernatant was adjusted to 100 µL. The sample concentration was adjusted according to the reaction results to make it within the range of the standard curve. Then, 1 mL of Sircol dye was added to the sample, followed by thorough mixing and centrifugation to obtain a collagen-dye complex. The supernatant was discarded to remove unbound dye, and 1 mL of a dye releasing agent was added to release the dye from the collagen-dye complex. Finally, the absorbance was quantitatively determined at 540 nm using a spectrophotometer. All measurements were performed two times, and the average value of triplicate samples was determined.

### Western blotting

CFs were washed with PBS (0.1 mol/L, pH 7-7.4) three times, lysed in a lysis buffer for 30 min at 4 °C, and then scraped off using a cell scraper before being mixed with a one-quarter volume of 5× loading buffer. The cell protein mixture was then boiled in a 100 °C water bath and cooled to room temperature. The protein concentration was determined using a Bradford protein assay kit (Amresco, M173-KIT). Equal amounts of protein (50 µg) were separated on 10% SDS-polyacrylamide gels and transferred onto nitrocellulose membranes. The strips were blocked with 5% nonfat milk for 1 h at room temperature. The strips were then incubated overnight with primary antibodies against AAT1 (Sigma, AV48205, 1:500), collagen I (Abcam, ab254113, 1:500), collagen III (Abcam, ab7778, 1:500), α-SMA (Abcam, ab5694, 1:500), β-catenin (Abcam, ab6302, 1:500), p-P38 MAPK (Abcam, ab4822, 1:500), P38 MAPK (Abcam, ab170099, 1:500) and GAPDH (Abcam, ab181602, 1:2000) diluted in PBS with 0.05% Tween 20 at 4 °C. The strips were rinsed using PBS with 0.05% Tween 20 for 30 min, followed by incubation with secondary antibodies for 1 h at room temperature. All protein bands were detected using Amersham ECL Western blotting detection reagents (GE Healthcare, RPN2106) and analyzed with a biological electrophoresis image analysis system.

### Quantitative real-time polymerase chain reaction

Total RNA was extracted from cells with TRIzol reagent (Takara, T9108) according to the manufacturer’s instructions. cDNA was synthesized using 800 ng of total RNA with a cDNA reverse transcription kit (CWBID, CW0741S). SYBR Premix Ex Taq™ (CWBIO, CW2602M) and a Roche real-time PCR detection system were used to quantify circRNA. The primer sequences for circNFIB were as follows: forward, 5’-tgaacgagatcaagcaccat-3’ and reverse, 5’-ctgctcggtgacag-3’. GAPDH was used as an internal control, and the primer sequences were as follows: forward, 5’-agccatgtacgtagccatcc-3’ and reverse: 5’-ctctcagcttggtggtgaa-3’.

### Statistical analysis

All experiments were repeated three times. The values are presented as the mean ± standard deviation. SPSS v19.0 was used for statistical analyses. One-way analysis of variance followed by post hoc analysis (Newman‒Keuls test) was used to compare differences among three or more groups. *P* < 0.05 indicated statistical significance.

## Results

### AAT1 overexpression inhibited excessive collagen production by TGF-β1-treated CFs

**Fig. 1:**
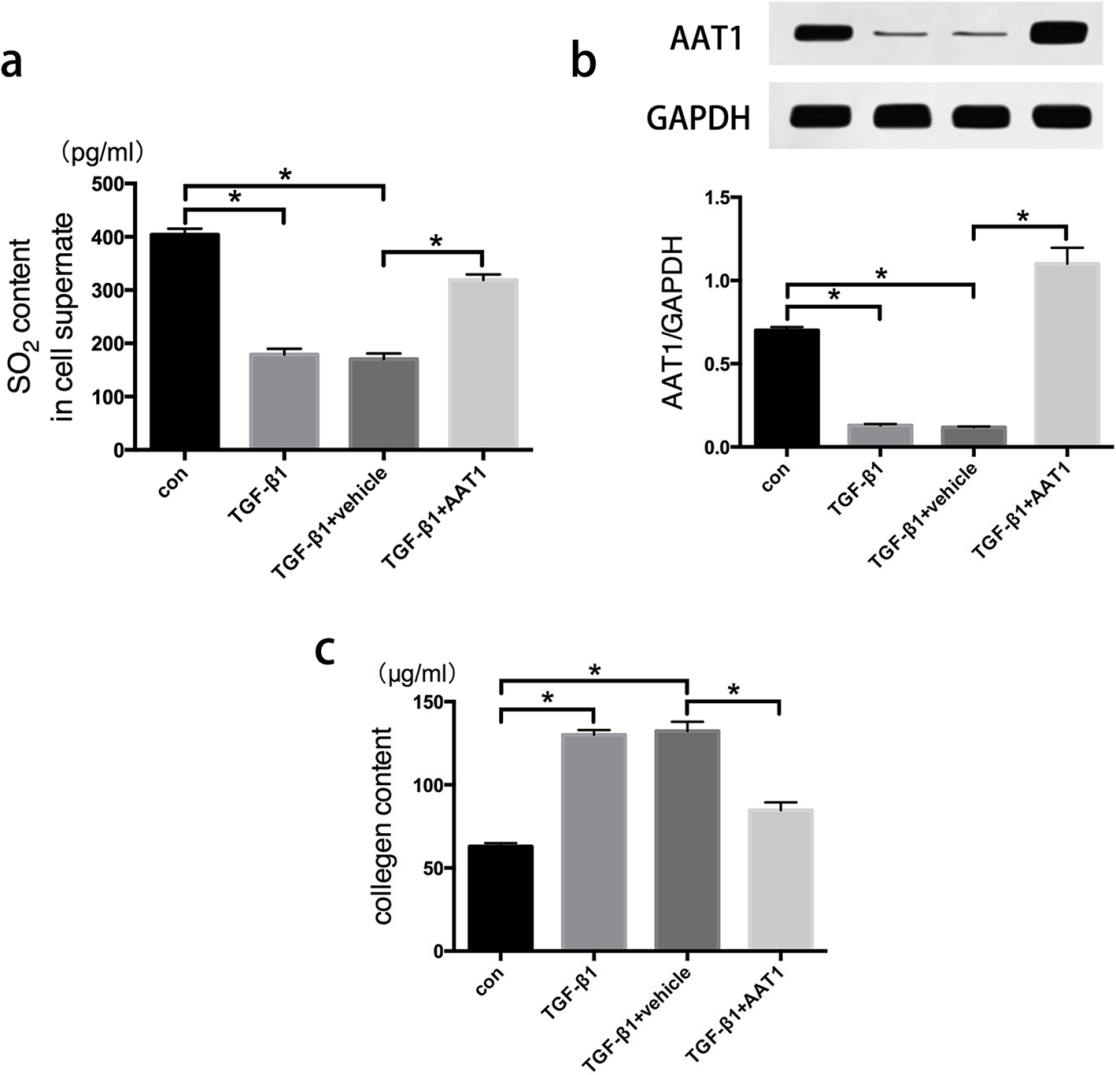
AAT1 overexpression inhibited excessive collagen production by TGF-β1-treated CFs. **a** Concentration of SO_2_ in cell supernatant of each group. **b** AAT1 protein expression in each group. **c** Concentration of collagen in cell supernatant of each group. * *P* < 0.05

To investigate the role of SO_2_/AAT1 in regulating TGF-β1-induced excessive collagen expression in CFs, we transfected a lentivirus vector containing cDNA encoding AAT1 into CFs to overexpress AAT1. As anticipated, in comparison with those in the vehicle group, the SO_2_ levels in the supernatant in the TGF-β1 group were markedly decreased, and AAT1 protein expression was upregulated in the AAT1 group. However, there was no difference between the AAT1 and TGF-β1 + AAT1 groups (Fig. 1 A-B). Furthermore, in control lentivirus-treated CFs, TGF-β1 significantly increased collagen levels in the supernatant. In comparison with those in the TGF-β1 group, collagen levels were decreased in the TGF-β1 + AAT1 group; however, no difference in collagen levels was observed between the AAT1 and TGF-β1 + AAT1 groups (Fig. 1 C).

Through these experiments, we successfully established a TGF-β1-induced myocardial fibrosis model in vitro and found that SO_2_/AAT1 could reverse the excessive production of collagen induced by TGF-β1.

### AAT1 overexpression inhibited excessive collagen production by CFs by upregulating circNFIB expression

**Fig. 2:**
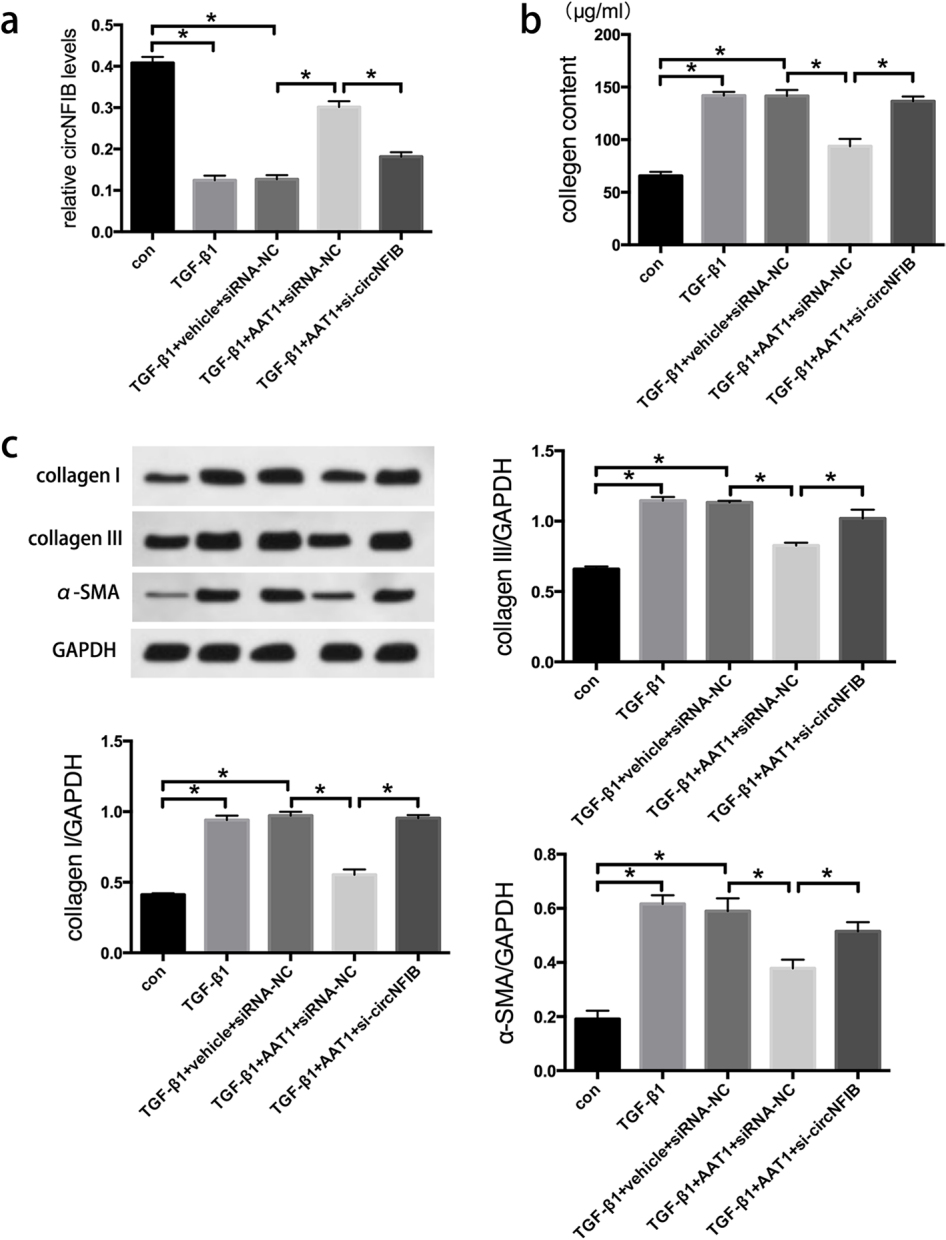
AAT1 overexpression inhibited excessive collagen production by CFs through upregulating circNFIB expression. **a** CircNFIB mRNA expression of each group. **b** Concentration of collagen in cell supernatant of each group. **c** Collagen I, collagen III and α-SMA protein expression in each group. * *P* < 0.05

We first measured the mRNA expression levels of circNFIB in each group. We found that the mRNA expression of circNFIB was significantly downregulated in the TGF-β1 group. In comparison with that in the TGF-β1 group, the mRNA expression of circNFIB was markedly increased in the TGF-β1 + AAT1 + siRNA-NC group (Fig. 2).

To further verify the role of circNFIB in alleviating myocardial fibrosis by endogenous SO_2_, we blocked circNFIB mRNA expression by transfecting siRNA into CFs. As anticipated, circNFIB mRNA expression was significantly downregulated in the TGF-β1 + AAT1 + si-circNFIB group, confirming the silencing efficiency of the siRNA (Fig. 2a). Moreover, we found that TGF-β1 significantly increased not only collagen levels in the supernatant but also collagen I, collagen III, and α-SMA protein levels in CFs. In comparison with those in the TGF-β1 group, collagen levels in the supernatant, as well as collagen I, collagen III, and α-SMA protein levels in CFs, were decreased in the TGF-β1 + AAT1 group. Furthermore, in comparison with those in the TGF-β1 + AAT1 group, these indicators were markedly decreased in the TGF-β1 + AAT1 + si-circNFIB group (Fig. 2b and c). Collectively, these results suggest that circNFIB upregulation is a key mechanism by which endogenous SO_2_ ameliorates myocardial fibrosis.

### CircNFIB alleviated TGF-β1-induced myocardial fibrosis by inhibiting the Wnt/β-catenin and p38 MAPK pathways in CFs

**Fig. 3:**
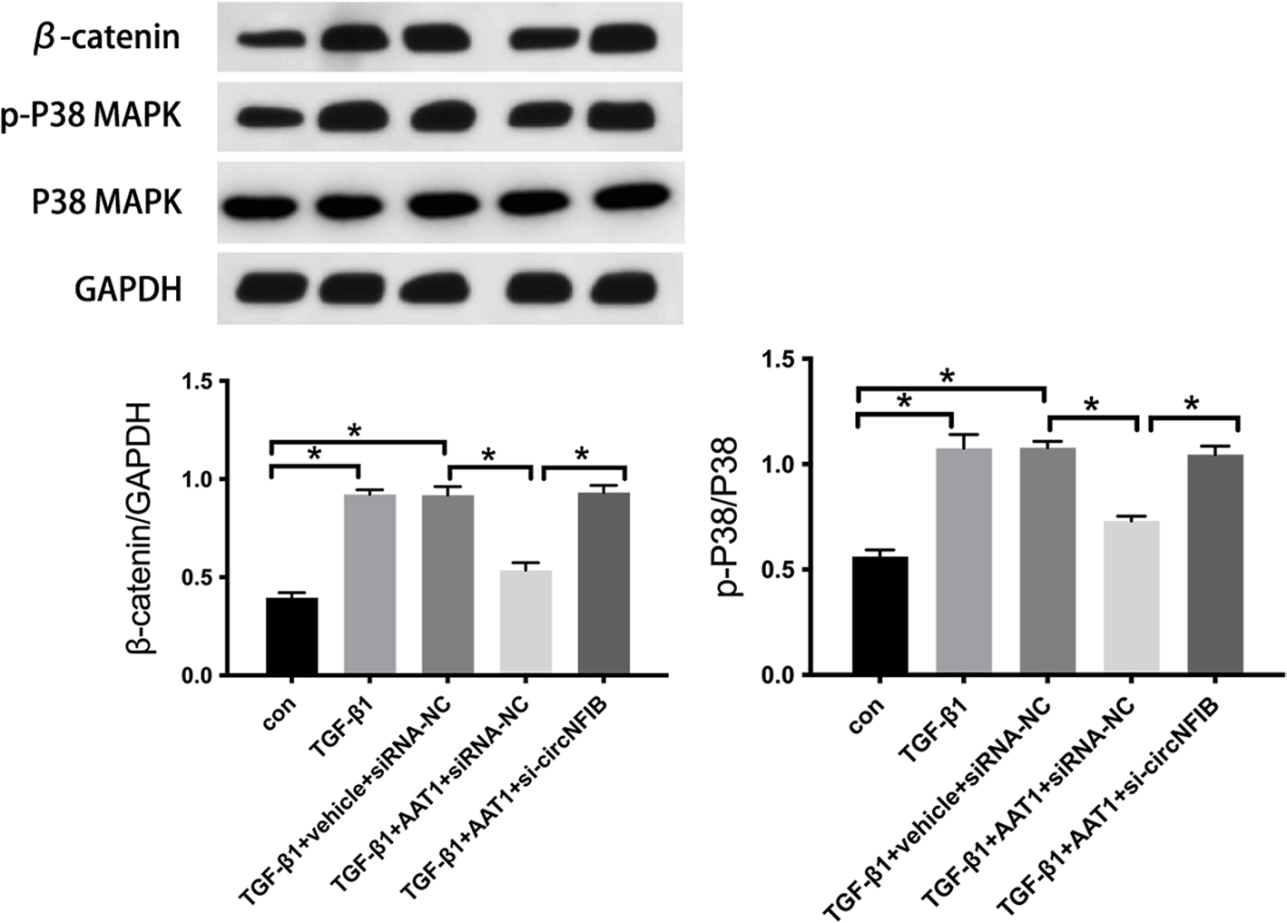
CircNFIB alleviated TGF-β1-induced myocardial fibrosis by inhibiting the Wnt/β-catenin and p38 MAPK pathways in CFs. * *P* < 0.05

To elucidate the mechanism by which circNFIB inhibited TGF-β1-induced excessive collagen production in CFs, we compared TGF-β1-induced β-catenin expression and P38 phosphorylation with or without AAT1 overexpression. In comparison with the vehicle group, the TGF-β1 group showed significantly higher β-catenin expression and P38 phosphorylation in CFs (*P* for both < 0.05; Fig. 3); however, in comparison with the TGF-β1 group, the TGF-β1 + AAT1 group showed significantly lower β-catenin expression and P38 phosphorylation (*P* for both < 0.05; Fig. 3). Furthermore, in comparison with the TGF-β1 + AAT1 + siRNA-NC group, the TGF-β1 + AAT1 + si-circNFIB group showed significantly higher β-catenin expression and P38 phosphorylation in CFs (*P* for both < 0.05; Fig. 3). These findings suggested that the Wnt/β-catenin and p38 MAPK pathways are involved in the mechanism by which circNFIB regulates TGF-β1-induced excessive collagen production in CFs.

## Discussion

Myocardial fibrosis is commonly involved in the occurrence and development of many heart diseases. In addition to acute causes, chronic causes such as aging, hypertension, valve disease, and cardiotoxic drugs can lead to myocardial fibrosis. These conditions can induce the production and accumulation of perivascular and interstitial collagen, affecting cardiac hemodynamic characteristics [13]. The main consequence of myocardial fibrosis is an increase in ventricular wall hardness, disturbance, and relaxation, leading to cardiac insufficiency. Myocardial fibrosis may also damage the conduction system of the heart, leading to atrial and ventricular arrhythmias [13, 14]. At present, clinical therapeutic strategies for myocardial fibrosis primarily include antihypertensive and anti-inflammatory therapy, lipid regulation, and cardioprotective drugs [15, 16]; however, many patients are not very sensitive to these treatment methods. Therefore, comprehensively investigating myocardial fibrosis occurrence and the underlying mechanisms is critical for developing better treatment strategies [17, 18].

Myocardial fibrosis is characterized by excessive collagen fiber deposition in the myocardium, enhanced collagen concentrations and volume fractions, imbalanced collagen proportions, and disordered arrangement. Type I collagen is present in most human tissues and is the most abundant protein in the human body, followed by type III collagen [19]. In the heart, fibroblasts and myofibroblasts are chiefly involved in type I and III collagen synthesis [20, 21]. Fibroblasts can differentiate into myofibroblasts under certain stimuli, and myofibroblasts reportedly have higher collagen production activity than fibroblasts [22–24]. SO_2_ is the fourth gas signal molecule after nitric oxide, carbon monoxide, and hydrogen sulfide. Endogenous SO_2_ can be produced by cardiomyocytes and participates in regulating several cardiovascular functions [25–30]. AAT, a key enzyme involved in endogenous SO_2_ production, is a pyridoxal phosphate-dependent aminotransferase that is divided into two subtypes. AAT1 is present in the cytoplasm, and AAT2 is present in mitochondria. AAT1 plays a major role in endogenous SO_2_ production. Although many studies have shown that endogenous SO_2_ can significantly improve myocardial fibrosis [8, 31], the specific mechanisms remain unclear.

CircRNAs are novel endogenous noncoding RNAs with a circular structure. They have garnered much attention because they are diverse, widely distributed, highly conserved, and stable, and they are not easily degradable by nucleases. CircRNAs participate in various physiological and pathological mechanisms in humans and are closely related to many diseases [32]. These factors play key roles in many cardiovascular diseases, including atherosclerosis, coronary heart disease, myocardial infarction, myocardial hypertrophy, heart failure, dilated cardiomyopathy, and arrhythmia [10, 31, 33, 34]; however, our understanding of their role in the pathogenesis of cardiovascular disease and organ fibrosis [13], including myocardial fibrosis, remains limited. CircRNA_010567 has been reported to promote myocardial fibrosis by suppressing miR-141 by targeting TGF-β1 [35]. A study reported that the expression of circRNA-000203 was markedly upregulated in the diabetic mouse myocardium and in angiotensin II-induced murine CFs. Furthermore, induced expression of circRNA_000203 enhanced the expression of fibrosis markers (collagen type I α2 chain, collagen type III α1 chain, and α-SMA) in murine CFs [36].

CircNFIB is derived from the exon regions of the *Nfib* gene. *Nfib* encodes nuclear factor 1 B-type (NF1-B), which is relatively highly expressed in the mouse heart and is involved in DNA replication, transcription and transcription regulation [11]. Du et al. found that circNFIB may be used as a biomarker for intrahepatic cholangiocarcinoma (ICC) patients, and the circNFIB-MEK-ERK axis may be a potential therapeutic target for ICC treatment [37]. In addition, a recent study reported that circNFIB plays a critical role in improving cardiac fibrosis in vivo and in vitro in an MI model [11]. In the present study, we found that circNFIB had a positive regulatory effect on myocardial fibrosis. Therefore, to some extent, we can expect this factor to be used as a diagnostic and prognostic biomarker of certain cardiovascular diseases in the future, which is also a problem that many researchers have been exploring [38–40]. This topic still requires further in-depth research on different disease models.

Does endogenous SO_2_ play a protective role in myocardial fibrosis by regulating circNFIB expression? In our in vitro TGF-β1-induced myocardial fibrosis model, circNFIB expression was significantly decreased, while AAT1 overexpression upregulated circNFIB expression and downregulated collagen expression. These results indicated that circNFIB might be involved in the process by which endogenous SO_2_ ameliorates myocardial fibrosis. To further verify this hypothesis, we transfected siRNA into cells with a lentivirus and then inhibited circNFIB expression. We found that downregulating circNFIB expression reversed the protective effect of endogenous SO_2_ against myocardial fibrosis.

Many studies have confirmed that various signaling pathways mediate tissue and organ fibrosis. The Wnt/β-catenin pathway, one of the most common signal transduction pathways, participates in regulating ventricular remodeling and myocardial fibrosis [41, 42]. Previous in vitro experiments revealed that Wnt5a activates CFs and induces IL-6 and TIMP-1 synthesis, thereby promoting the occurrence of myocarditis and myocardial fibrosis [43]. Moreover, in vivo and in vitro, blocking the Wnt signaling pathway with a PORCN inhibitor (WNT-974) could alleviate myocardial fibrosis and improve cardiac function [44]. P38 MAPK pathway activation is also involved in myocardial fibrosis. It is well known that extracellular matrix accumulation principally results from increased synthesis and decreased degradation of type I and III collagen, decreased metalloproteinase activity, and increased tissue metalloproteinase inhibitor activity [45]. Studies have shown that p38 MAPK increases the expression of various metalloproteinases in CFs through diverse mechanisms [46, 47] and reduces that of tissue metalloproteinase inhibitor-1 [48]. Moreover, p38 MAPK has been shown to reduce type I and III collagen expression [49–51], consequently playing a role in myocardial fibrosis regulation.

Previous studies have shown that circNFIB overexpression inhibits the activation of the p38 MAPK pathway, alleviating myocardial fibrosis. This could be related to its role as an endogenous sponge of miR-433 [11]. To examine the mechanism through which circNFIB alleviates myocardial fibrosis, we assessed β-catenin and p-p38 expression in cells in each group. We observed that circNFIB upregulation by AAT1 overexpression alleviated TGF-β1-induced myocardial fibrosis by inhibiting the Wnt/β-catenin and p38 MAPK pathways; the activation of these pathways was significantly inhibited after siRNA-mediated silencing of circNFIB expression. These findings suggest that endogenous SO_2_ inhibits the Wnt/β-catenin and p38 MAPK pathways by upregulating the expression of circNFIB, thereby playing a crucial role in ameliorating myocardial fibrosis. We propose the existence of a “SO_2_–circNFIB–Wnt/β-catenin and p38 MAPK–myocardial fibrosis” axis. However, how does endogenous SO_2_ regulate circNFIB expression? How does circNFIB inhibit its downstream signaling pathway? There are still many targets and mechanisms in this regulatory axis that warrant further investigation.

## Conclusion

We found that circNFIB expression was significantly downregulated in TGF-β1-treated myocardial fibroblasts. Furthermore, endogenous SO_2_ upregulated circNFIB expression, which alleviated myocardial fibrosis by inhibiting the Wnt/β-catenin and p38 MAPK signaling pathways. The concept of a “SO_2_–circNFIB–Wnt/β-catenin and p38 MAPK–myocardial fibrosis” axis is of key theoretical significance to identify target and related mechanisms through which endogenous SO_2_ alleviates myocardial fibrosis and provides an important scientific basis for clinical diagnosis and treatment in the future.

## Supplementary Information

**Additional file 1:** **Supplementary Figure 1.** The original and uncropped gel image of AAT1.

**Additional file 2:** **Supplementary Figure 2.** The original and uncropped gel image of collagen I and β-catenin.

**Additional file 3:** **Supplementary Figure 3.** The original and uncropped gel image of collagen III and α-SMA.

**Additional file 4:** **Supplementary Figure 4.** The original and uncropped gel image of GAPDH.

**Additional file 5:** **Supplementary Figure 5.** The original and uncropped gel image of p-P38 and GAPDH.

## Data Availability

The datasets used and/or analysed during the current study available from the corresponding author on reasonable request.

## Abbreviations

SO_2_: sulfur dioxide
AAT1: aspartate aminotransferase 1
TGF-β1: transforming growth factor-β1
circRNA: Circular RNA
CFs: cardiac fibroblasts
MAPK: mitogen-activated protein kinase
