# Supplementary figures and images for: Elucidating the role of circNFIB in myocardial fibrosis alleviation by endogenous sulfur dioxide
Source: BMC Cardiovasc Disord. 2022 Nov 20;22:492. doi: 10.1186/s12872-022-02909-x (PMC9677687; doi:10.1186/s12872-022-02909-x)

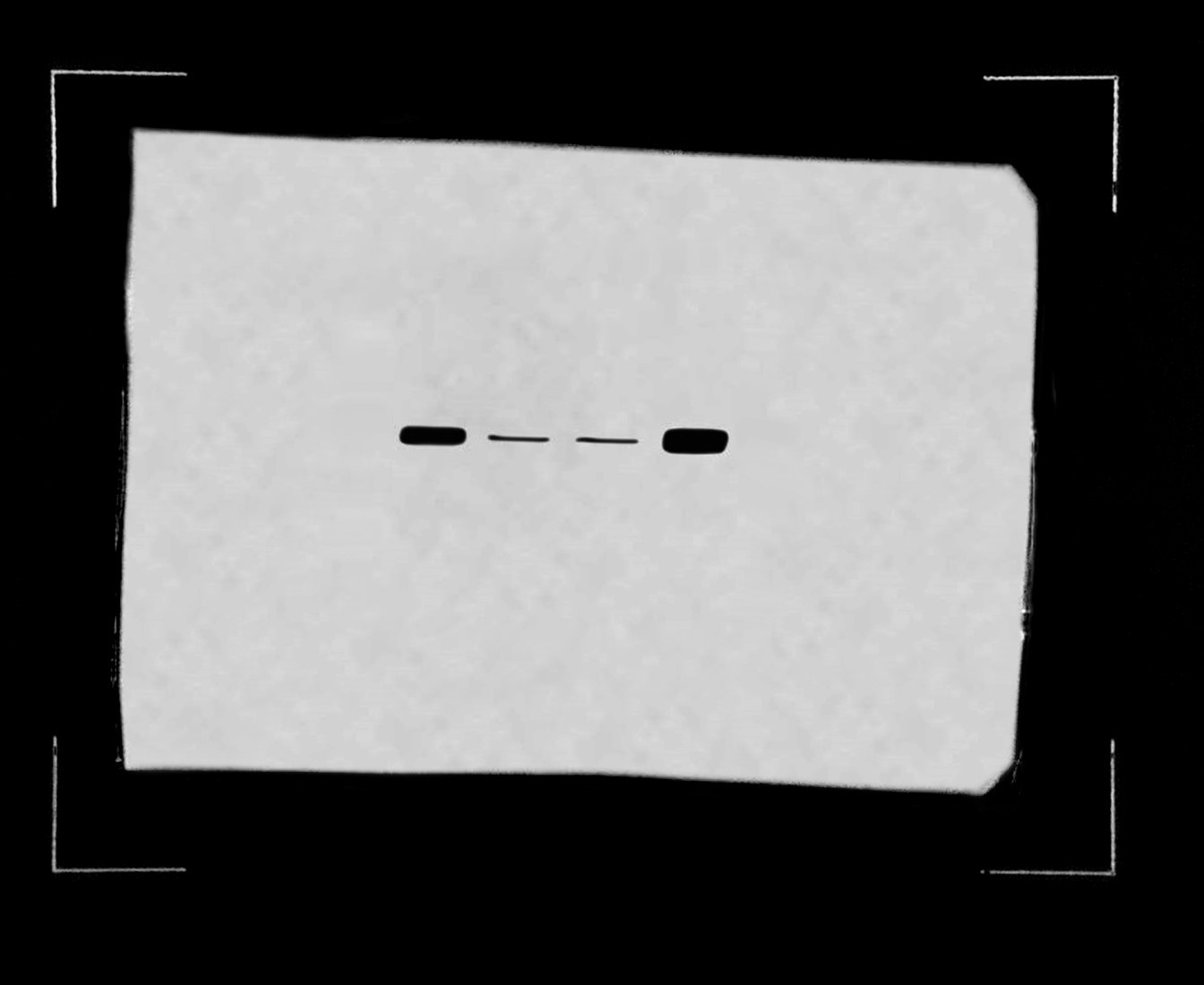

Supplement: Supplementary file 1 — Additional file 1: Supplementary Figure 1. The original and uncropped gel image of AAT1. [file 12872_2022_2909_MOESM1_ESM.jpg]

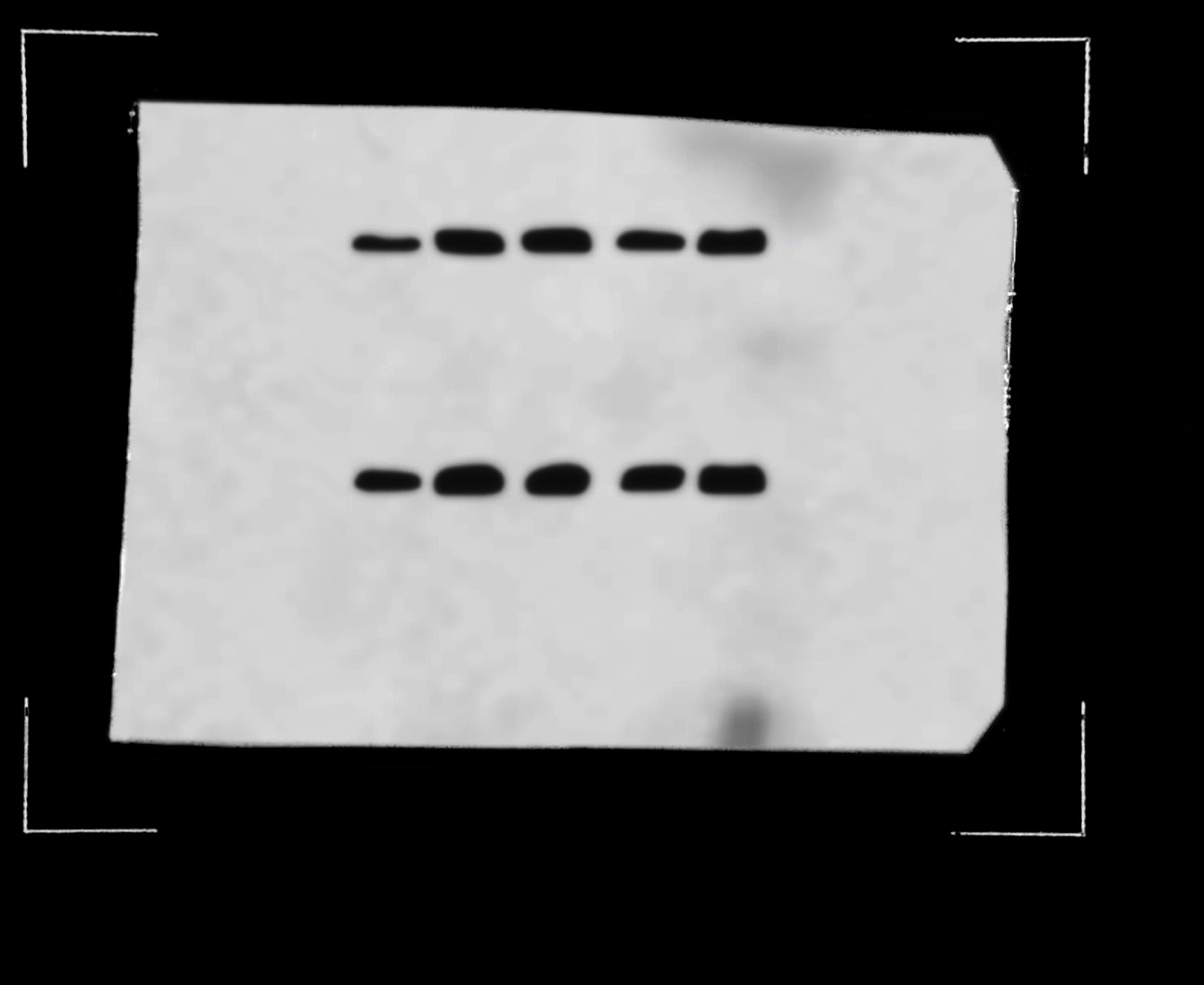

Supplement: Supplementary file 2 — Additional file 2: Supplementary Figure 2. The original and uncropped gel image of collagen I and β-catenin. [file 12872_2022_2909_MOESM2_ESM.jpg]

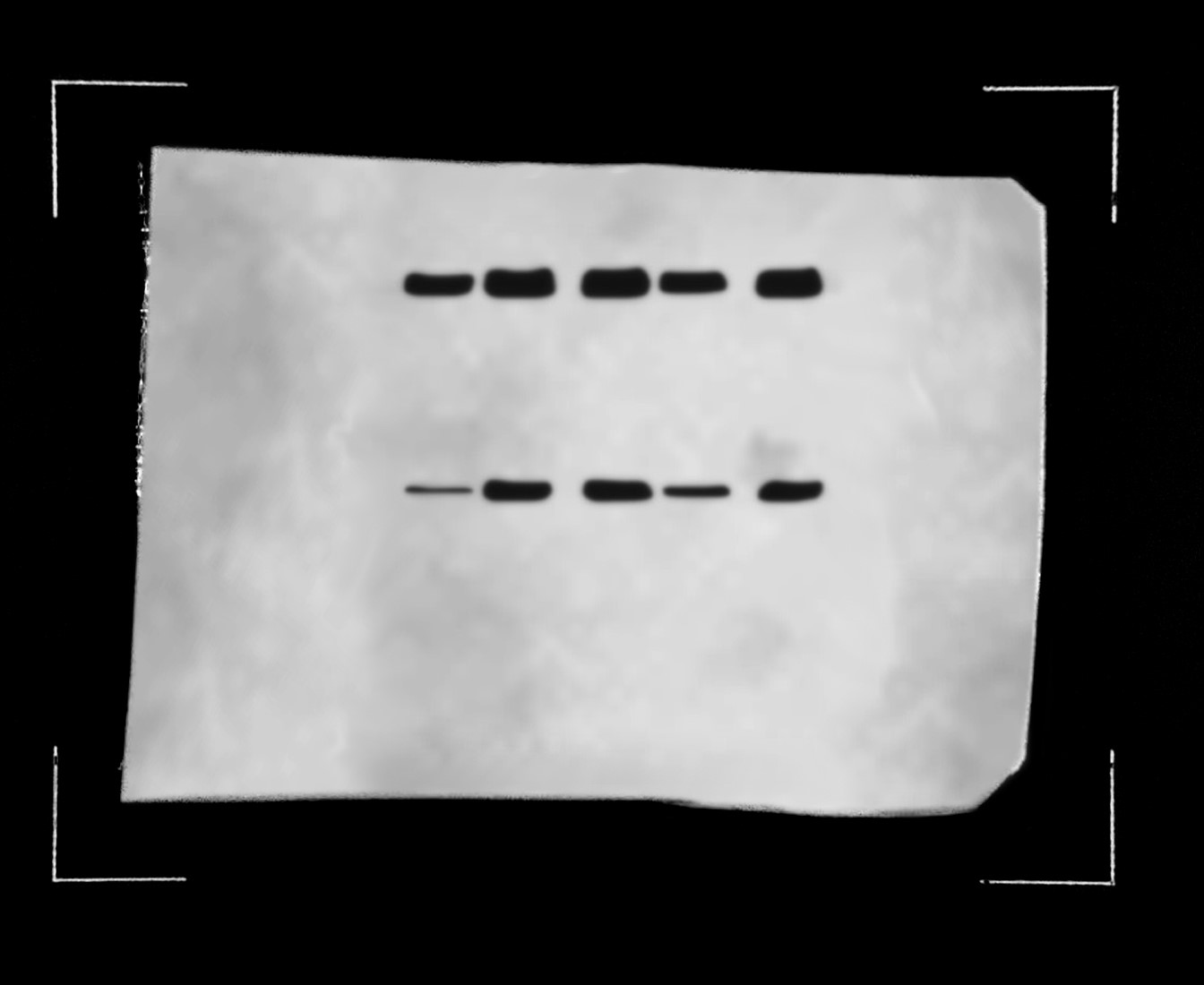

Supplement: Supplementary file 3 — Additional file 3: Supplementary Figure 3. The original and uncropped gel image of collagen III and α-SMA. [file 12872_2022_2909_MOESM3_ESM.jpg]

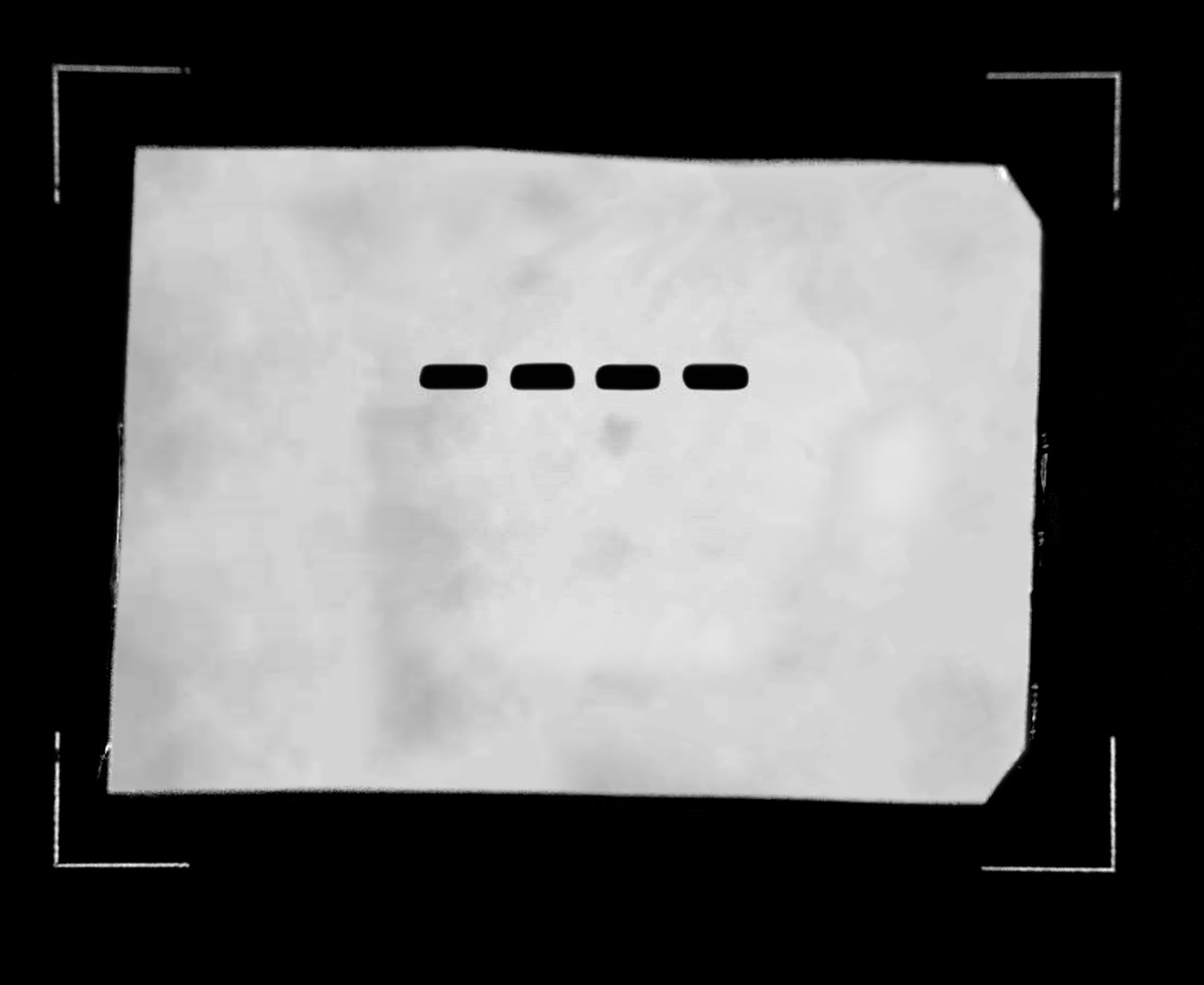

Supplement: Supplementary file 4 — Additional file 4: Supplementary Figure 4. The original and uncropped gel image of GAPDH. [file 12872_2022_2909_MOESM4_ESM.jpg]

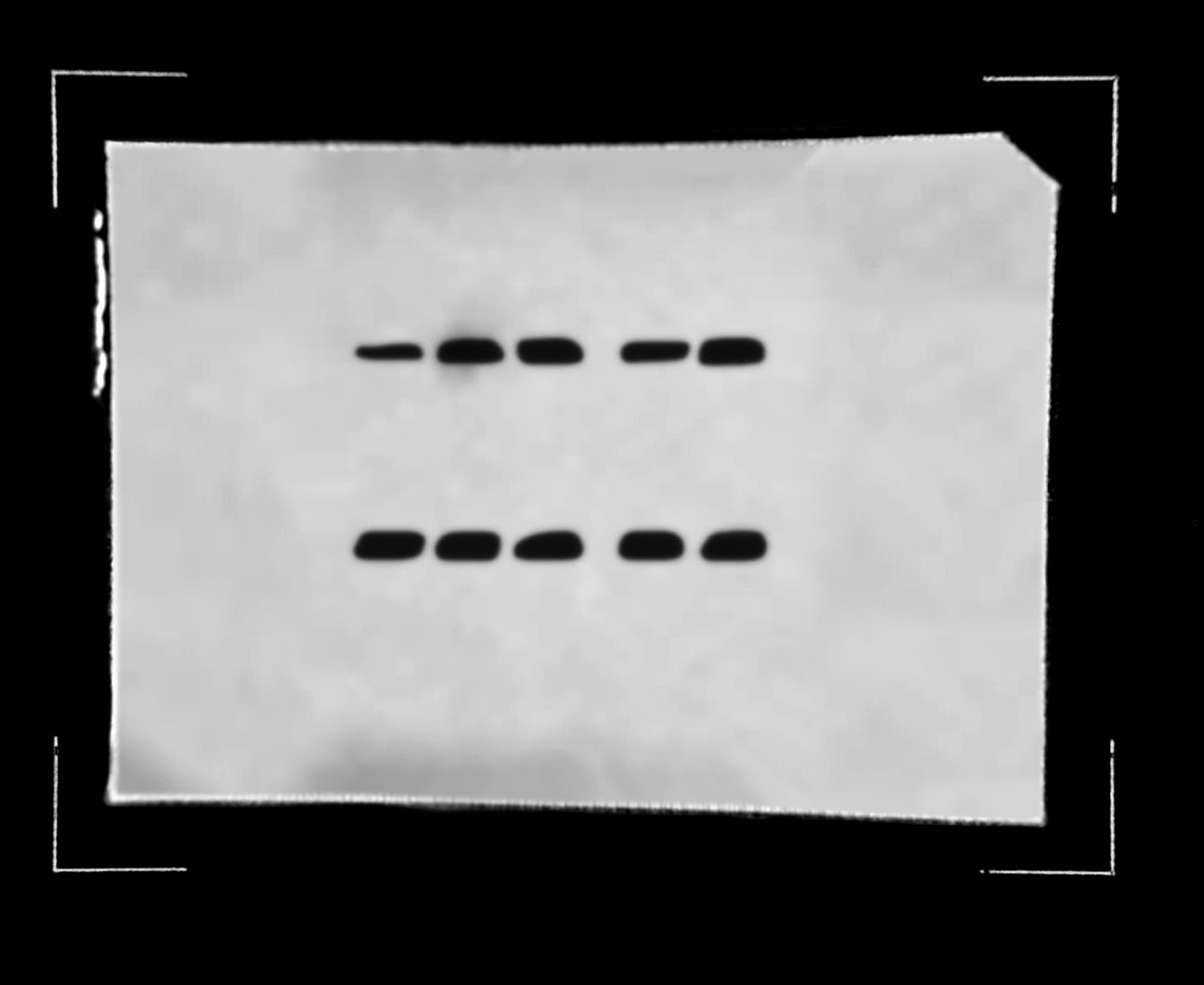

Supplement: Supplementary file 5 — Additional file 5: Supplementary Figure 5. The original and uncropped gel image of p-P38 and GAPDH. [file 12872_2022_2909_MOESM5_ESM.jpg]
